# QuickStats

**Published:** 2014-02-21

**Authors:** FROM THE NATIONAL CENTER FOR HEALTH STATISTICS

**Figure:**
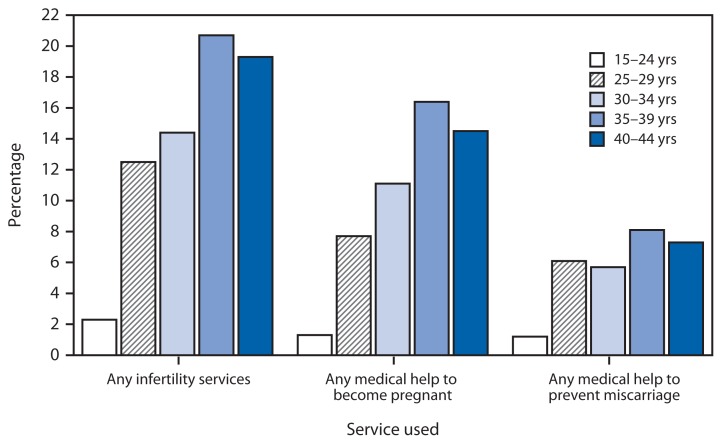
Percentage of Females Aged 15–44 Years Ever Using Infertility Services,* by Age Group — National Survey of Family Growth, United States, 2006–2010 * Infertility services include medical help to become pregnant or to prevent miscarriage; both types of medical help could be reported.

During 2006–2010, among females aged 15–44 years, the percentage of those who ever used infertility services increased through age 34 years, leveling off for women aged 35–44 years. Approximately one fifth of women aged 35–39 years and 40–44 years had ever used infertility services, either to become pregnant or to prevent a miscarriage, compared with 2.3% among females aged 15–24 years. Use of medical help to become pregnant ranged from 1.2% for females aged 15–24 years to 16.4% for women aged 35–39 years. Use of medical help to prevent miscarriage showed a similar but less steep increase with age.

**Source:** Chandra A, Copen CE, Stephen EH. Infertility service use in the United States: data from the National Survey of Family Growth, 1982–2010. Natl Health Stat Rep 2014(73).

**Reported by:** Anjani Chandra, PhD, 301-458-4138, achandra@cdc.gov; Casey Copen, PhD.

